# Neural Plasticity in Common Forms of Chronic Headaches

**DOI:** 10.1155/2015/205985

**Published:** 2015-08-20

**Authors:** Tzu-Hsien Lai, Ekaterina Protsenko, Yu-Chen Cheng, Marco L. Loggia, Gianluca Coppola, Wei-Ta Chen

**Affiliations:** ^1^Department of Neurology, National Yang-Ming University School of Medicine, Taipei 112, Taiwan; ^2^Section of Neurology, Department of Internal Medicine, Far Eastern Memorial Hospital, New Taipei City 220, Taiwan; ^3^MGH/MIT/HMS Athinoula A. Martinos Center for Biomedical Imaging, Massachusetts General Hospital, Harvard Medical School, Charlestown, MA 02129, USA; ^4^Department of Neurophysiology of Vision and Neurophthalmology, G.B. Bietti Foundation-IRCCS, 00198 Rome, Italy; ^5^Institute of Brain Science, National Yang-Ming University School of Medicine, Taipei 112, Taiwan; ^6^Neurological Institute, Taipei Veterans General Hospital, Taipei 112, Taiwan

## Abstract

Headaches are universal experiences and among the most common disorders. While headache may be physiological in the acute setting, it can become a pathological and persistent condition. The mechanisms underlying the transition from episodic to chronic pain have been the subject of intense study. Using physiological and imaging methods, researchers have identified a number of different forms of neural plasticity associated with migraine and other headaches, including peripheral and central sensitization, and alterations in the endogenous mechanisms of pain modulation. While these changes have been proposed to contribute to headache and pain chronification, some findings are likely the results of repetitive noxious stimulation, such as atrophy of brain areas involved in pain perception and modulation. In this review, we provide a narrative overview of recent advances on the neuroimaging, electrophysiological and genetic aspects of neural plasticity associated with the most common forms of chronic headaches, including migraine, cluster headache, tension-type headache, and medication overuse headache.

## 1. Introduction

In its 2010 Global Burden of Disease Survey, the World Health Organization reported tension-type headache (TTH) and migraine as the second (20.1%) and third (14.7%) most prevalent disorders in the world, exceeded only by dental caries [1]. An earlier meta-analysis of 107 studies reported the one-year prevalence of headaches among adults to be a staggering 46%, with TTH (42%), migraine (11%), and chronic daily headache (3%) standing out as the most common types [2]. With such statistics, headache has taken its place among the disorders plaguing the global population.

While such a commonplace condition may be easily dismissed, the impact of headache is not to be taken lightly. Using “years living with disability” as a measure of disease impact, the WHO rated migraine as the 7th most disabling of all 289 diseases surveyed (excluding the nonspecific “other musculoskeletal disorders”) [1]. In conditions such as migraine and other headaches, the chronicity of the disorder itself may have profound negative impacts. Compared to episodic headache, chronic headache has been associated with more disability, reduced quality of life, and greater direct and indirect economic losses [3, 4]. While the definitions of chronic headaches will vary with their subtypes, as will their individual impacts, the great personal toll associated with chronic headache is well illustrated by chronic daily headache, whose sufferers experience headache for ≥15 days per month, for >3 months [5].

Unfortunately, the mechanisms responsible for the development of chronic headaches remain unknown. Barring rare exceptions such as “new daily persistent headache,” most patients with chronic headaches initially experience only episodic attacks [6]. While it is still unclear why some individuals go on to develop chronicity, epidemiological studies report a number of risk factors, including obesity, history of frequent headache (>one per week), overuse of analgesics (>10 or 15 days/month), and caffeine consumption, among others [7]. As the search for a mechanism continues, numerous hypotheses have been put forth regarding the role of neural plasticity in headache chronification.

Thanks to recent advances in electrophysiology and neuroimaging, now we are able to test these hypotheses directly on the human brain. In this review, we first provide a narrative overview of the most common forms of chronic headaches and then discuss the neural plasticity underlying specific headache disorders by comparing available electrophysiological and neuroimaging studies in their episodic and chronic forms, according to the tools used and the hypotheses proposed. Emphasis will be laid on migraine as it is the most well studied type of these headaches.

## 2. Common Forms of Chronic Headache

### 2.1. Chronic Migraine

Although it is not the most common of the headache disorders we will discuss, migraine headache is by far the most disabling and most researched condition [1]. According to the definitions put forth by the International Classification of Headache Disorder, migraine consists of recurrent unilateral throbbing headache attacks of moderate to severe intensity that are aggravated by physical activity [8]. Associated symptoms include nausea, vomiting, and hypersensitivity to light and/or sound [8]. Patients with migraine could have aura, a transient visual, sensory, or other central nervous system symptoms before or concurrent with headache [8]. While most patients experience infrequent episodic attacks (episodic migraine, EM), some patients may have chronic migraine (CM) defined as any headaches for ≥15 days per month and headache with the above migraine characteristics for ≥8 days per month, for >3 months [8]. Although the mechanisms underlying migraine headache remain debated, there is likely an interaction between genetic predisposition and environmental factors at work [9]. These two components together may be responsible for increased overall migraine susceptibility and for the development of cortical spreading depression—the electrical phenomenon underlying migraine aura [9].

### 2.2. Chronic Cluster Headache

CH is a primary headache disorder characterized by severe, strictly unilateral pain, lasting from 15 to 180 minutes [8]. Accompanying these attacks, patients usually experience ipsilateral cranial autonomic symptoms (tearing, conjunctive ejection, nasal congestion, rhinorrhea, forehead sweating, miosis, ptosis, and ear fullness) and a feeling of agitation. Unlike most other forms of headache, CH tends to occur in “bouts,” with patients experiencing regular and frequent attacks for a period of time. However, as many as 21% of patients develop chronic CH, they suffer from at least one CH per month for at least one year. Interestingly, the prevalence of chronic CH appears to be low in Asians [10].

### 2.3. Chronic Tension-Type Headache

TTH is the most common type of headache [11]. Unlike migraine, TTH is a “featureless” headache—usually mild, bilateral, nonpulsatile (pressing), and not aggravated by daily activities. Common migraine-associated symptoms, including nausea and vomiting, are usually absent in TTH, although mild photo- or phonophobia may be present [11]. As a result of its comparatively mild severity, TTH has remained out of the medical and research spotlight. Nonetheless, it has been found to significantly increase healthcare utilization and work absence rates and consequently remains costly to society [12]. Moreover, like migraine, TTH can evolve from its episodic form to a chronic condition (chronic TTH, defined as ≥15 headache days/month). Chronic TTH is extremely disabling and is regarded as one of the most neglected and difficult to treat forms of headache [13].

### 2.4. Medication Overuse Headache

MOH results from regular overuse of abortive medication, exceeding 10 or 15 days per month (depending on the analgesic), for more than 3 months [8]. While MOH is defined as a distinct secondary headache syndrome, it is also commonly associated with primary headaches. While the prevalence of MOH in the general population is only 1-2% according to epidemiological studies, it may in fact be the most common type of headache to present in specialty clinics [55]. MOH is a recognized risk factor for headache evolution, especially when resulting from the use of barbiturates and triptans [6, 7].

## 3. Electrophysiological Evidence of Neural Plasticity in Chronic Headaches

### 3.1. Central Sensitization

Sensitization of the trigeminal pain network (i.e., even beyond the first-order neuron) has been proposed to underlie migraine pathophysiology. As shown by the animal studies conducted by Burstein and colleagues, applying brief chemical stimulation with inflammatory agents to the dura in rats led to peripheral sensitization of the first-order neurons in the dorsal root ganglia of C2/C3 and trigeminal ganglion and central sensitization of the second order neurons in the trigeminal nucleus caudalis (also known as trigeminocervical complex). As a result, the rats treated with inflammatory agents had increased excitability in response to brush or nonnoxious heat stimulation [56–58]. This same sensitization, in a subset of migraine patients who experience extracranial pain, may extend to third order neurons in the thalamus as well [59]. In support of this, further work from the same group provides evidence of central sensitization in the thalamus, both in the rat model described above and in findings of exaggerated thalamic functional MRI (fMRI) activation in human migraineurs experiencing cutaneous allodynia during ictal periods [60].

Electrophysiological studies of trigeminal processing also characterize neural plasticity in association with CH [61], although the findings are diverse and sometimes conflicting. While the classic blink reflex has repeatedly shown no signs of sensitization [62, 63], a number of other studies suggest CH is accompanied by a general sensitization of pain processing [14]. Researchers have found evidence of faster rates of R2 blink reflex recovery after supraorbital paired pulse electric stimulation, perhaps indicating reduced central opioid activity [64]. Studies have also reported increased excitability of trigeminal processing on the affected side of the head compared to the unaffected side [65–68]. Moreover, studies have consistently shown reduced thresholds of pressure pain, electric pain, nociceptive corneal reflex, and RIII reflex on the symptomatic side relative to the asymptomatic side, in both episodic and chronic CH [62, 69–71].

In TTH, evidence is mounting in support of a neural plasticity characterized by central sensitization. An intriguing study in patients with chronic TTH showed increased suprathreshold pain sensitivity both in skin and in muscle, and in both cephalic and extracephalic regions [72]. This generalized hyperalgesia implicated central sensitization as an underlying mechanism. Consistent with these findings, another event-related potentials study showed that painful CO_2_ laser stimulation over the pericranial zone leads to a higher amplitude of the N2a-P2 complex together with a higher degree of pericranial muscle tenderness in patients with chronic TTH than in controls [73].

Neural plasticity in the pathogenesis of MOH has also been linked to sensitization. Using SSEP, studies have shown an increase in the amplitude of painful and nonpainful cortical responses, with the latter normalizing following recovery from MOH [20, 21, 74]. These abnormalities in cortical responses to somatosensory stimulation seem to be strongly influenced by genetic factors and the types of medications being overused [74, 75]. Sensitization has also been observed at the level of the spinal cord, where the activity of the endocannabinoid system was reported to be enhanced in patients with MOH and normalized after detoxification [76, 77]. Animals treated with analgesics as a model of MOH demonstrated increased pain perception, as manifested in terms of lower withdrawal reflex thresholds [78]. This change could be gradually reversed after stopping drug infusion, but a different study showed that sensitization may persist even after drug discontinuation [79].

### 3.2. Habituation

Habituation refers to “a response decrement as a result of repeated stimulation” [80], and patients with migraine often show a “lack of habituation,” that is, no decrease—or even an increase—in response following repetitive stimulation. These migraine-related deficits in the normal habituation phenomenon have been most thoroughly examined using the method of visual evoked potentials (VEP), although similar findings have been reported with a number of other methods, including somatosensory and auditory evoked potentials, blink reflex, and laser evoked potentials [14]. Interestingly, the defective habituation appears to normalize immediately before or during a migraine attack (preictal/ictal periods). In patients with CM, the habituation pattern during interictal periods is similar to that during a migraine attack, indicating CM as a status of never-ending migraine [15, 74, 81–83].

Research also suggests that changes in habituation may be associated with the transition from EM to CM. Habituation studies using nonpainful somatosensory evoked potentials (SSEP) have reported similarities between the electrophysiological patterns of ictal EM and CM, including initial excessive cortical activation followed by normal habituation during stimulus repetition [20]. Aurora and colleagues have shown reduced visual suppression to transcranial magnetic stimulation (TMS) in patients with EM, which becomes more severe with CM (Table 1) [18, 19]. Consistent with these findings, we have used magnetoencephalography (MEG) to demonstrate that CM patients consistently show patterns of cortical excitability similar to those of ictal EM [16] and provide evidence of plasticity associated with CM remission back to interictal EM (Table 1) [17]. The mechanisms underlying interictal deficits in habituation and the associated changes accompanying migraine chronification remain largely unknown. In general, both habituation and sensitization may result from repeated stimulation, and it has therefore been proposed that these two opposing processes compete to determine the final response [84]. Consistent with this hypothesis, an imbalance between inhibitory and excitatory cortical mechanisms—perhaps primary or secondary to abnormal thalamic control, which is due in turn to hypoactive aminergic projections from the brainstem—has been proposed as the culprit in the abnormal habituation response (for a review see [14]).

A lack of habituation in the blink reflex on the affected side has been observed in episodic CH patients [65, 85, 86]. The habituation slope was positively correlated with the number of days since the onset of the CH bout and with the daily attack frequency [86].

Moreover, MOH patients have also shown deficient habituation mechanisms during contingent negative variation [87] and laser-evoked potential [88] recordings and dysfunction of the inhibitory circuits by TMS [89].

### 3.3. Defective Endogenous Pain Modulation

The role of the central nervous system in pain is not limited to the processing of nociception from the periphery to the higher order regions of the brain; rather, the central nervous system is capable of actively modulating pain perception through descending pain modulatory mechanisms. Among the oldest theories of central inhibition, spinal gate control theory posits a top-down mechanism operating from the cortex to modulate the responses of dorsal horn neurons in the spinal cord [90]. Diffuse noxious inhibitory control (DNIC), also known as conditioned pain modulation in humans, is an excellent example of this type of descending modulation [91]. Originally characterized in rats, DNIC refers to the phenomenon by which the application of a tonic painful conditioning stimulus results in an inhibition of dorsal horn neurons and associated sensory and motor responses [92]. Dysfunction of this network (i.e., disinhibition) has been observed in various pain disorders [93–97], and may therefore represent a pathophysiological mechanism underlying pain disorders of different etiology.

To date, a number of studies have shown vastly altered endogenous pain modulation in migraine patients. In the first exploration of DNIC dysfunction associated with migraine, researchers used the cold pressor test as a conditioning stimulus and assessed the nociceptive flexion reflex [98]. As expected, healthy volunteers experienced significant inhibition of the nociceptive flexion reflex during the cold pressor test. On the other hand, patients suffering migraine and chronic TTH showed facilitation, rather than inhibition, of the nociceptive flexion reflex, thereby indicating dysfunction in the systems underlying DNIC [98]. Similar findings have been reported in migraine patients, especially those with medication overuse (MO) [76].

As we saw with habituation, changes in DNIC may also be associated with the transformation from EM to CM. Using capsaicin as a conditioning stimulus, research has shown increased R2 area of the blink reflex in CM sufferers, as compared to their EM (with aura) counterparts [99]. The results suggest that patients with CM experience more severe dysfunction of the DNIC than those who only experience occasional attacks. However, it is worth noting that negative results have also been reported in studies of DNIC changes associated with migraine, suggesting that the changes may be quite subtle [100, 101].

## 4. Neuroimaging Evidence of Neural Plasticity in Chronic Headaches

### 4.1. Functional Neuroimaging

Functional neuroimaging has played a remarkable role in elucidating the pathophysiology of migraine, from demonstrating that hypoperfusion and cortical spreading depression are the underlying mechanisms of visual aura [102, 103] to suggesting that the brainstem may be a “migraine generator” [104–106]. The majority of these studies have been designed to capture the activity of the brain during migraine attacks, that is, during the ictal period. Nonetheless, a number of studies have investigated patients' brain responses to painful and other stimuli during interictal periods. They have consistently observed increased activation of a network of brain regions collectively known as the “pain-matrix” (i.e., a term, now progressively running out of favor, traditionally used to describe a collection of regions activated by painful stimulation, and including the primary and secondary somatosensory cortices, anterior cingulate cortex, insula, prefrontal cortex, the thalamus and others [107]). Decreased activation can be vice versa observed in areas responsible for pain inhibition (e.g., pons, ventral medulla), thereby suggesting an imbalance between facilitation and inhibition likely resulting from maladaptive neural plasticity (for review, see [29]).

Significant efforts have also been made to link migraine to abnormalities in functional connectivity measured by resting-state fMRI (rs-fMRI). Unlike experiments examining brain responses to stimuli (including both experimentally applied stimulation and clinical pain), functional connectivity experiments instead look for concurrent fluctuations of the fMRI signals during a task-free rest period [108]. This line of research has revealed patterns of aberrant functional connectivity in patients with migraine and has also noted an association between degree of connectivity and clinical variables, such as headache frequency and disease duration [29]. For instance, a rs-fMRI study has revealed that with increasing frequency of monthly migraine attacks the periaqueductal gray, a key node in the descending pain modulatory system [109–111], becomes more functionally connected to pain processing regions (e.g., insula and secondary somatosensory cortex) and less functionally connected to pain-modulatory regions (e.g., orbitofrontal cortex) [112]. Similar to the results from stimulus- or task-related fMRI, these resting state studies also suggest that dysfunctional dynamics between pain modulatory and pronociceptive regions may be implicated in the pathophysiology of migraine.

While some of the abovementioned experiments have been conducted exclusively with CM patients, many of the studies in the literature have been limited by their inclusion of both chronic and episodic migraine patients. Among those that have focused on CM, a combined electrophysiology and PET study noted increased cerebral metabolism in the brainstem [19]. A rs-fMRI study showed that functional connectivity with affective pain regions differed between CM patients and controls in a number of regions, including the anterior insula, amygdala, pulvinar, mediodorsal thalamus, middle temporal cortex, and periaqueductal gray [30]. It was further found that connectivity in some of these regions correlated with disease duration.

Just like migraine, recent years have seen many neuroimaging studies probing the etiology of episodic and chronic CH. Early PET studies provided evidence of activation in the ipsilateral hypothalamus, contralateral thalamus, anterior cingulate cortex, and bilateral insulae in CH [113, 114], with activation of the hypothalamus appearing to be specifically associated with pain attacks [115]. Similar patterns of neural response have been confirmed across imaging modalities, with fMRI studies reporting activation of the hypothalamus, prefrontal cortex, anterior cingulate cortex, contralateral thalamus, ipsilateral basal ganglia, insula, and bilateral cerebellum during bouts of CH [116]. Finally, functional imaging has also demonstrated alterations in functional connectivity, including at the level of the primary visual network and between hypothalamus and its connections with frontal, occipital, and cerebellar areas in CH patients [117–119].

Neuroimaging studies have provided valuable information on neural plasticity underlying MOH as well. An early PET study provided evidence of hypometabolism in the orbitofrontal cortex that persisted after detoxification, implying that the dysfunction observed is the cause, rather than a consequence, of MOH [120]. An fMRI study reported hypoactivation of the right supramarginal gyrus and the right inferior and superior parietal cortex, all of which recovered near normal patterns of activation six months after medication withdrawal [121]. These areas may be involved in pain modulation. Another fMRI study showed dysfunction in the mesocorticolimbic dopamine circuit, including the ventromedial prefrontal cortex and the substantia nigra/ventral tegmental area complex [122]. Dysfunction in the ventromedial prefrontal cortex was reversible following withdrawal, while dysfunction in the substantia nigra/ventral tegmental area complex remained persistent. The results suggest that the mechanisms of MOH may involve the dopaminergic reward system, similar to other chronic pain disorders such as fibromyalgia [97, 123].

### 4.2. Structural Neuroimaging

#### 4.2.1. Gray Matter: Voxel- and Surface-Based Morphometry Studies

Studies from a wide variety of chronic pain conditions, including chronic back pain [124], fibromyalgia [125–128], rheumatoid arthritis [129], menstrual pain [130], and vulvar pain [131], indicate that long term exposure to pain might cause structural alterations in a number of brain regions [132]. The most common approach to assessing gray matter structure is that of voxel-based morphometry (VBM). Except for the very first publication to apply the method to headache (which reported no change), VBM studies have consistently found decreases in gray matter volume in multiple brain areas that broadly overlap with the “pain-matrix” [31–37]. Several studies have further provided evidence that within migraine sufferers, gray matter volume may change dynamically between ictal and interictal periods [133] and that gray matter volume changes more broadly correlate with attack frequency [34–36]. As with functional imaging, these findings are limited by the studies' focus on episodic migraine. Only two of the studies described here recruited any CM patients without history of MO, with relatively small sample sizes (11 and 3 patients each) [33, 35].

The mechanisms underlying structural changes in the brain associated with migraine remain to be determined. Based simply on the similarity of findings between migraine studies and those of other headache subtypes and other pain disorders, it has been proposed that these changes are consequences rather than causes of repeated attacks [134]. One potential mechanism underlying the effect of continuous exposure to pain on structural integrity may be neuroinflammation. Using integrated positron emission tomography/magnetic resonance imaging (PET/MRI), Loggia et al. have observed evidence of neuroinflammation/glial activation in the brain of chronic low back pain, most prominently in the thalamus [135]. As the thalamus was shown to exhibit reduced gray matter volume in the same patient population [124], it is possible that the excessive production of cytokines and other proinflammatory chemicals released by activated glia [136] represents pathophysiological mechanisms responsible for the structural alterations observed in chronic pain conditions. However, future studies will need to investigate whether glial activation is observed in other pain disorders, including migraine.

Another technique used to evaluate structural alteration in patients with migraine and other pain disorders is surface-based morphometry, a technique that allows the measurement of cortical thickness instead of volume [137]. It is worth noting that, while cortical thinning and reductions in gray matter density/volume are more commonly reported in chronic pain disorders, increases in structural metrics have also been described, such as in fibromyalgia and in chronic vulvar pain [127, 131].

In line with these observations, two early studies by Hadjikhani and colleagues reported increased, rather than decreased, cortical thickness in the somatosensory and visual motion areas in patients with EM [38, 39]. A subsequent study by Maleki et al. examined potential differences between patients with low (1-2 days/month) and high (8–14 days/month) headache frequencies [41]. The group reported reduced cortical thickness in the low frequency group and increased cortical thickness in the high frequency group. Other researchers have found mixed results, reporting both increases and decreases in cortical thickness in different brain regions, as well as different directions in the association between cortical thickness and clinical parameters, such as disease duration and attack frequency [42, 43]. It is also worth noting that, while changes in cortical thickness are a normal part of ageing, the correlations between cortical thickness, cutaneous pain threshold, and age are atypical in episodic migraine patients [138, 139]. Again, the biological mechanisms underlying these structural changes and their relation with clinical and psychophysical parameters need to be elucidated and may reflect the differential contributions of various neuroinflammatory processes, such as swelling, gliosis, excitotoxicity-mediated necrosis, and others.

Structural imaging studies of CH also have had mixed results, disagreeing not only on the brain regions affected, but even on the direction of grey matter volume changes. Using VBM, a pioneering structural imaging study revealed gray matter volume increases in the bilateral posterior hypothalamus, a region colocalized with the functional changes observed in PET imaging of CH [140]. Examining episodic CH patients during pain-free “out-bout” periods, other studies have suggested gray matter volume decreases in areas associated with the pain-matrix, including the thalamus, anterior cingulate cortex, insula, basal ganglia, cingulum, and frontal cortex [141, 142]. Others still have shown evidence of dynamic alterations in gray matter volume in regions such as the temporal lobe, hippocampus, insular cortex, and cerebellum [143]. These regions have been implicated in a number of pain related functions, including attention and emotion regulation, fear conditioning, and nociceptive encoding during pain perception and processing. Furthermore, in patients with chronic CH, gray matter decreases were noted in multiple areas involved in pain modulation [143]. The same study reported negative correlations of gray matter volume with disease duration and positive correlations with attack interval, indicating that gray matter undergoes dynamic and reversible changes during the various stages of CH.

Reversibility of morphological changes has been reported in other chronic pain conditions, including a recent surface-based morphometry study [144] demonstrating that successful treatment in patients with chronic back pain may reverse not only abnormal brain function, but also abnormal brain structure: after treatment, patients had increased cortical thickness in the left dorsolateral prefrontal cortex, which was thinner before treatment compared to controls. This observation again suggests that brain plasticity in response to pain is bidirectional.

Decreases in gray matter volume of pain-related brain structures were also reported in patients with chronic TTH, including anterior cingulate cortex, insula, orbitofrontal cortex, parahippocampal gyrus, and dorsal rostral pons [145]. These decreases were positively correlated with headache duration, which, as the authors suggest, may be an indication of structural changes being the consequence of central sensitization.

In MOH patients with migraine history, significant gray matter volume decreases were reported in the orbitofrontal cortex, anterior cingulate cortex, insula and precuneus, as well as volume increases in the periaqueductal gray, thalamus and ventral striatum [115]. The same group later published posttreatment results, reporting that the gray matter volume increases in the midbrain returned to baseline only in the subset of patients experiencing clinical improvement. Low gray matter volume in the orbitofrontal cortex was also associated with poor treatment response [146]. A recent study explored both functional connectivity and morphological changes in MOH patients with migraine, using EM patients and healthy volunteers as controls [147]. The authors reported no structural differences in group comparisons but did identify negative correlations between migraine duration and gray matter volume in the frontal regions, precuneus, and hippocampus.

#### 4.2.2. White Matter

While the clinical significance is unclear, migraine patients have well-documented white matter hyperintensities [148]. To explore these white matter changes, most studies have adopted the method of diffusion tensor imaging (DTI). DTI allows for visualization of the orientation and anisotropy of water diffusion, which in turn allows for detection of microstructural alterations of white matter not visible by conventional MRI [53]. Studies using different methods (e.g., histogram-based analysis, region-of-interest, or tract-based spatial statistics), exploring both whole brain and targeted brain areas, have reported changes in various DTI parameters [36, 44–50]. As with gray matter studies, it remains unknown whether these changes contribute to headache chronification or are merely consequences of headache. Dynamic alterations in thalamic microstructure (higher fractional anisotropy in the interictal phase, which normalized during the ictal phase) have been reported, perhaps reflecting plastic peri-ictal modifications of local fibers [51]. To date, only one study has recruited patients with CM, but the results showed no changes in any DTI parameters in patients with CM or EM, as compared to healthy controls [52]. However, 15 (71%) of the 21 CM patients reported concomitant MO, potentially confounding results. In addition to migraine, DTI studies have provided evidence of changes in patients with CH regarding white matter diffusivity throughout the brain, including the pain-matrix [141, 149–151].

### 4.3. Biochemical Neuroimaging

Another promising line of research in the study of the neural mechanisms underlying chronic headache or pain disorders is represented by the use of magnetic resonance spectroscopy (MRS). MRS allows noninvasive and in vivo exploration of the molecular composition of tissue, by identifying certain metabolites involved in physiological or pathological processes. By using techniques such as single voxel spectroscopy or chemical shift imaging, researchers were able to reveal the presence of biochemical alterations in the brain of patients with various chronic pain disorders. For instance, studies have demonstrated a reduced concentration of N-Acetylaspartate (NAA), a marker of neuronal integrity in chronic low back pain [152–156], complex regional pain syndrome [157], fibromyalgia [158–160], and neuropathic pain patients [161–163], in various brain regions. Other studies have revealed increases in the concentration of the excitatory neurotransmitter glutamate (Glu), or of a combination of glutamate/glutamine in fibromyalgia [158, 164, 165], but decreases in chronic low back pain patients [156].

Despite the great potential, not many MRS studies have been conducted in migraine patients. Of those that have been conducted, the majority focus on disturbed energy metabolism, indicating a possible role of mitochondrial dysfunction in migraine pathophysiology [166]. Another study has revealed decreases in NAA and glutamate and increases in the concentration of myoinositol, in the cerebellar vermis of patients with familial hemiplegic migraine type 1 [167]. Our group has compared ^1^H-MRS metabolite ratios in EM and CM patients [168]. Patients with EM presented with the elevated NAA/creatine (Cr) ratios at the dorsal pons, indicating possible neuronal hypertrophy. On the contrary, CM patients had NAA/Cr levels similar to those of healthy controls. The NAA/Cr ratios were inversely correlated with headache frequency and intensity. We propose that the repetitive noxious stimuli might pose a detrimental effect leading to the neuronal loss of this region during migraine evolution from EM to CM.

## 5. Genetic Aspects of Neural Plasticity in Chronic Headaches

Genes may influence the cerebral processes that lead to the progression from episodic to chronic headache and determine distinctive morphofunctional properties [169]. Most studies in the genetic aspect of neural plasticity associated with chronic headaches focused on migraine and MOH.

Though few in number, studies have been conducted to examine whether select gene polymorphisms might influence neural plasticity (habituation/sensitization) in MOH. The angiotensin-converting enzyme D/D genotype appears to serve as an influencing factor in migraine attack frequency [170], as well as in substance abuse behavior [171, 172]. Di Lorenzo et al. [75] found that D/D carriers presented with the highest grand averaged SSEP amplitudes, reflecting sensitization, and the most severe deficits in habituation, although other MOH patients overall did not habituate either. This abnormal electrophysiological pattern gradually disappeared in the D/I and I/I carriers, in whom the cortical response habituated normally. Moreover, the group has observed that angiotensin-converting enzyme polymorphisms influence overuse behavior: in patients carrying the D/D genotype, more prolonged MOH is associated with greater sensitization and greater habituation deficits [75].

Considering that MOH bears resemblance to an abuse disorder and that previously identified susceptibility genes, such as the angiotensin-converting enzyme polymorphism, have also been linked to substance abuse behavior, researchers have sought to examine whether there are psychiatric differences between MOH sufferers with various polymorphisms. Researchers have examined whether the brain-derived neurotrophic factor (BDNF) Val66Met and wolframin His611Arg (WFSI) polymorphisms, both being linked to psychiatric illness and dependence behavior, might be related also to MOH. They observed that individuals carrying the RR WFSI [173] and the non-G/G BDNF [174] genotypes showed significantly higher monthly drug consumption than non-R/R WFSI and G/G BDNF carriers. On the same notion, Terrazzino et al. [175] found that MOH patients carrying the 516T serotonin 5HT2A receptor gene polymorphism, but not that of A-1438G, have significantly higher monthly drug consumption than their 516CC counterparts.

Others have taken an epigenetic approach with the hopes of assessing whether gene expression patterns may change along with patients' migraine state. Hershey et al. [176, 177] found unique gene expression patterns in the subset of MOH patients who responded to analgesic detoxification. Gene ontology indicated that many of the identified genes are involved in cell signaling pathways, phosphorylation of cellular components, and immunological pathways [177].

Genetic linkage studies have reported a significant association between CM and the long allele of monoamine oxidase A 30 bp VNTR and CYP1A2^*∗*^1F variant, both enzymes responsible for triptan degradation [178]. The latter variant was found to be significantly associated with triptan overuse and drug response within MOH patients [179]. Knowing the important role of dopamine brain circuitry in drug dependence behavior, researchers have assessed the role of dopamine metabolism-related genes on susceptibility to MOH [180]. Allele 10 of the dopamine transporter gene was significantly underrepresented in patients with MOH when compared with episodic migraine sufferers. However, a recently published candidate-gene association study failed to find any significant associations between high-frequency-to-chronic migraine and the 144 single-nucleotide polymorphisms previously implicated in migraine or identified as interesting secondary hits in genome-wide association studies [181].

In summary, inheritance appears to play an important role in determining predisposition to specific clinical manifestations of migraine and the progression to CM, especially when related to MOH. Furthermore, the association between gene polymorphisms and characteristic neurophysiologic patterns in CM suggests that genetics can influence the way the brain responds plastically to chronic head pain and excessive drug consumption.

## 6. The Link between Neural Plasticity and Headache Chronification

To date electrophysiological and neuroimaging studies have revealed different aspects of neural plasticity associated with chronic headaches, especially migraine. Table 1 compares these study findings between EM and CM to characterize the neural plasticity associated with migraine chronification. Of note, migraine chronification involves various aspects of neural plasticity in pain-related neural networks. Despite inconclusive findings in brain structures, earlier studies have characterized neural plasticity in association with CM evolution by brain excitability change (central sensitization, habituation change, impaired inhibition), altered biochemistry and metabolism, and aberrant functional connectivity. Some studies (please refer to MEG and LEP in Table 1) further suggest an ictal-like response pattern in interictal periods of CM. Taken together, it is assumed that chronic headache may be an abnormal functional status of never-ending headache underpinned by neural plastic responses to recurrent headaches. Genetic predisposition, as discussed above, may influence the evolution of chronic headaches. However, the true genetic effect upon neural plasticity can only be disentangled if the complex interaction between genes and electrophysiology or neuroimaging can be clarified in future longitudinal studies.

Other common forms of chronic headaches, such as chronic CH and chronic TTH and MOH, also share some features of neural plasticity with CM, notably changes in brain excitability (Table 1). Nevertheless, it remains undetermined whether these common features of neural plasticity can be regarded as neurologic signatures for chronic headaches. It is unknown either whether there are headache-specific neural plasticity that may help differentiate between chronic headaches or develop mechanism-based therapy against chronic headaches.

## 7. Conclusion

Neural responses to episodic headache are initially adaptive and physiologic but later become maladaptive and pathologic, eventually creating a vicious cycle resulting in chronic headache. This process of headache evolution is associated with neural plasticity in brain excitability, biochemistry, function, and even structures. Genetic factors are likely to contribute to this process. Further studies are needed to elucidate if there are common features of neural plasticity among various chronic headaches that may serve as neurologic signatures for chronic headaches, or conversely, headache-specific neural plasticity that may help in the diagnosis and treatment of different chronic headaches.

## Figures and Tables

**Table 1:** Neural plasticity in episodic and chronic migraine, without medication overuse.

	Episodic migraine (EM)	Chronic migraine (CM)
Electrophysiology		
VEP	Lack of habituation and peri-ictal normalization [14]	No specific study
MEG	Peri-ictal normalization of visual cortical excitability, reflecting a dynamic modulation of cortical activities [15]	Persistent ictal-like visual cortex excitability [16]; in patients who remitted from CM to EM, the MEG pattern shifted to that characterizing EM between attacks, that is, decreased initial amplitude with subsequent deficient habituation [17]
TMS	Hyperexcitability measured by TMS indices of phosphene thresholds and magnetic suppression of perceptual accuracy [18]	Reduced visual suppression correlating with high cortical excitability [18, 19]
SSEP	Abnormal habituation during interictal period and central sensitization (increase of N20-P25 amplitude) during ictal period [14]	Increase of N20-P25 amplitudes recorded interictally in patients with CM compared with in patients with EM, indicating excessive cortical activation of the somatosensory pathway [20]
BAEP	Lack of habituation of wave IV-V, especially with symptomatic vertigo [14]	No specific study
LEP	Lack of habituation of N1 (generated by secondary somatosensory cortex) and N2-P2 (generated by ACC and insula) during interictal and ictal periods Sensitization represented by increased N2-P2 amplitude in the ipsilateral headache side during ictal period [14]	Increase of amplitudes and rostral shift within ACC in patients with CM, similar to EM patients in the ictal period [21, 22]

Neuroimaging		
Functional		
PET	Activation of certain brain areas during ictal period indicating the involvement of specific brain areas associated with various symptoms in migraine including photophobia, nausea, and vertigo [23–26] Ligand PET: changes of serotonin and opioid receptors and activities, indicating possible roles these neurotransmitters play and related neural plasticity associated with migraine [27, 28]	Increased cerebral metabolism at brainstem compared to the global flow and also decreased cerebral metabolism in the medial frontal, parietal, and somatosensory cortex, indicating a potential dysfunction in the inhibitory pathways [19]
fMRI	Greater activation of pain-matrix areas and less activation of pain inhibition areas [29]	No specific study
rs-fMRI	Aberrant functional connectivity mostly in pain-matrix and also involving different networks including salience, default mode, central-executive, somatomotor, and frontoparietal attention networks [29]	Aberrant functional connectivity in affective pain regions including anterior insula, amygdala, pulvinar, mediodorsal thalamus, middle temporal cortex, and periaqueductal gray [30]
Structural		
VBM	Decrease of gay matter volume of multiple brain areas within pain-matrix [31–37]	No specific study; only two studies recruited small numbers of CM patients (11 and 3 patients each) without definite conclusions [33, 35]
SBM	Increase thickness of the somatosensory cortex and visual motion areas [38, 39]; no changes [40]; thickness of somatosensory cortex decrease in low frequency (1-2 days/month) and increase in high frequency (8–14 days/month) [41]; mixed results of increase and decrease of cortical thickness in other brain areas [42, 43]	No specific study
DTI	Changes of white matter microstructures in areas such as corpus callosum and cingulate gyrus [36, 44–50] Dynamic alteration of fractional anisotropy noted at thalami, in relation with peri-ictal/ictal status [51]	No changes in one study recruiting both CM and EM patients [52]
Biochemical		
MRS	Higher NAA/Cr ratio at dorsal pons, indicating possible neuronal hypertrophy; inverse correlation with headache frequency and intensity [53] Changes of the excitatory glutamate in the ACC and insula, indicating [54]	Lower NAA/Cr as compared with EM with inverse correlation with headache frequency and intensity, indicating possible progression of neuronal loss during evolution [53]

VEP: visual evoked potential, MEG: magnetoencephalography, TMS: transcranial magnetic stimulation, SSEP: somatosensory evoked potential, BAEP: brainstem auditory evoked potential, LEP: laser evoked potential, ACC: anterior cingulate cortex, PET: positron emission topography, fMRI: functional magnetic resonance imaging, rs-fMRI: resting state functional magnetic resonance imaging; VBM: voxel-based morphometry, SBM: surface-based morphometry, DTI: diffusion tensor imaging, MRS: magnetic resonance spectroscopy, and NAA/Cr: N-acetylaspartate/creatine.
